# Mapping tissue microstructure of brain white matter in vivo in health and disease using diffusion MRI

**DOI:** 10.1162/imag_a_00102

**Published:** 2024-03-06

**Authors:** Ying Liao, Santiago Coelho, Jenny Chen, Benjamin Ades-Aron, Michelle Pang, Valentin Stepanov, Ricardo Osorio, Timothy Shepherd, Yvonne W. Lui, Dmitry S. Novikov, Els Fieremans

**Affiliations:** Bernard and Irene Schwartz Center for Biomedical Imaging, Department of Radiology, New York University Grossman School of Medicine, New York, NY, United States; Center for Advanced Imaging Innovation and Research (CAI ^2^R), Department of Radiology, New York University Grossman School of Medicine, New York, NY, United States; John A. Burns School of Medicine, University of Hawaii at Manoa, Honolulu, HI, United States; Department of Psychiatry, New York University Grossman School of Medicine, New York, NY, United States

**Keywords:** diffusion MRI, microstructure imaging, white matter, early development, ischemia, multiple sclerosis

## Abstract

Diffusion magnetic resonance imaging offers unique *in vivo* sensitivity to tissue microstructure in brain white matter, which undergoes significant changes during development and is compromised in virtually every neurological disorder. Yet, the challenge is to develop biomarkers that are specific to micrometer-scale cellular features in a human MRI scan of a few minutes. Here, we quantify the sensitivity and specificity of a multicompartment diffusion modeling framework to the density, orientation, and integrity of axons. We demonstrate that using a machine learning-based estimator, our biophysical model captures the morphological changes of axons in early development, acute ischemia, and multiple sclerosis (total N = 821). The methodology of microstructure mapping is widely applicable in clinical settings and in large imaging consortium data to study development, aging, and pathology.

## 1 Introduction

Diffusion magnetic resonance imaging (dMRI) is a non-invasive technique that maps the probability density S(t,x) of water molecules’ displacements x(t) in each imaging voxel (Jones, 2010). With typical displacements $\sqrt{\langle x^{2}\left(t\right)\rangle }\sim 10\;\mu$m during diffusion times t~50  ms used in the clinic, the dMRI signal becomes uniquely sensitive to how tissue structure on the micrometer scale restricts the diffusion of water molecules, opening a window into cellular-level details such as cell density, shape, orientation, and permeability of cell membranes (Alexander et al., 2019; Novikov et al., 2019). Thanks to this unique *in vivo* contrast, dMRI is particularly promising in detecting microstructural changes related to developmental and pathological processes in the brain white matter (WM), including myelination and demyelination, axonal growth and axonal loss, pruning, beading, and inflammation (Horsfield & Jones, 2002).

The greatest technical challenge of clinical dMRI is to uncover the exact relationship between cellular-level features and the dMRI signal—that is, to make dMRI not just sensitive, but also specific to tissue microstructure. This would turn an empirical diagnostic technique into a quantitative and reproducible scientific measurement paradigm, enabling improved understanding of the changes that underlie development, aging, and disease, and tracking of its progression. So far, widely adopted dMRI techniques, such as diffusion tensor imaging (DTI) (Basser et al., 1994) and diffusion kurtosis imaging (DKI) (Jensen et al., 2005), offer ways to represent the dMRI signal S(t,q) (the Fourier transform of the displacements probability density S(t,x)) as expansions up to $q^{2}$ and $q^{4}$, correspondingly. However, these empirical signal representations inherently lack specificity to cellular-level phenomena, as they do not rely on any assumptions about tissue microgeometry.

**Fig. 1.**
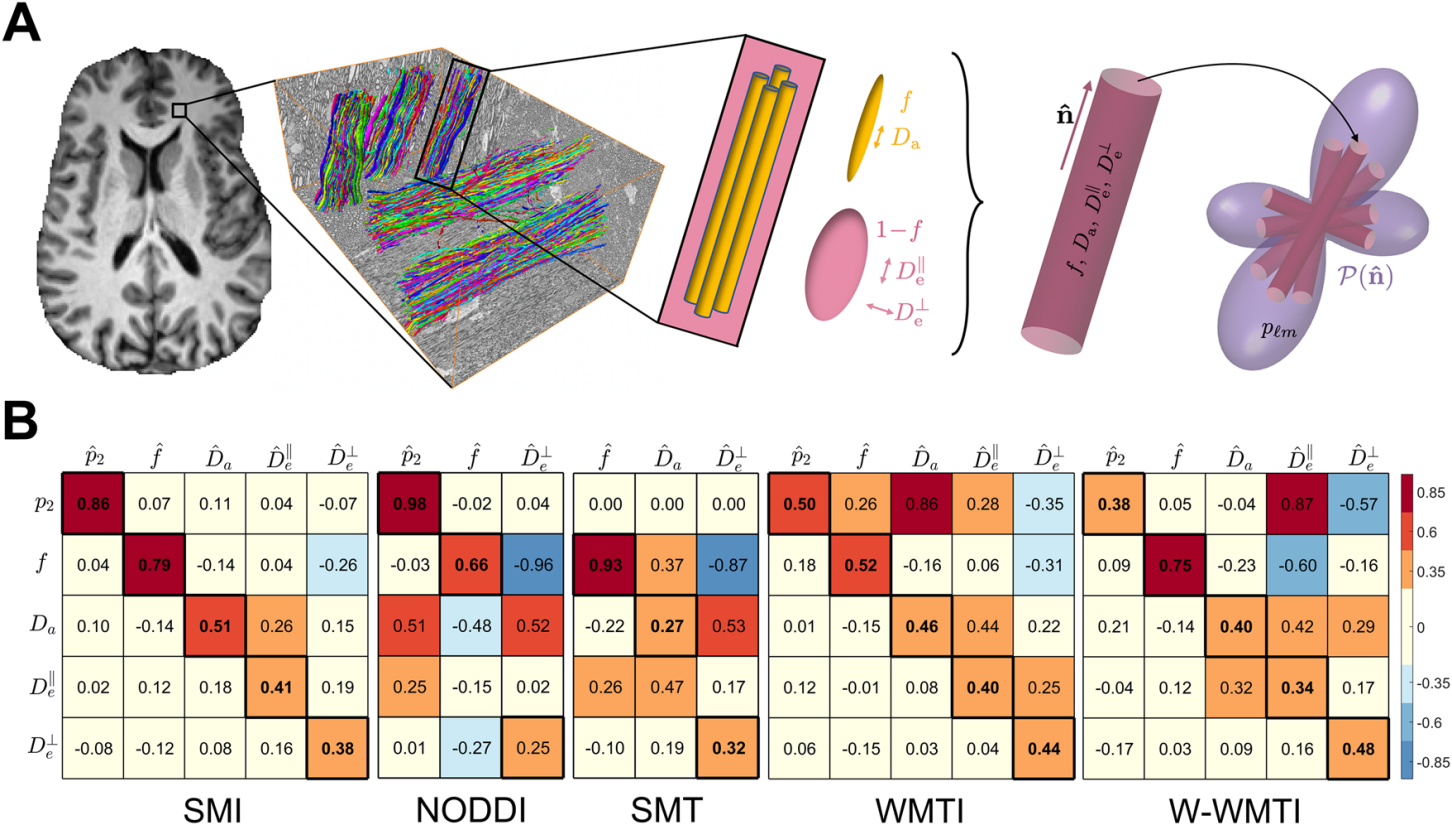
Sensitivity and specificity matrix of SM estimators. (A) Elementary fiber fascicles of the SM, consisting of the IAS and EAS, are described by at least four independent parameters: f, $D_{a}$, $D_{e}^{∥}$, $D_{e}^{⊥}$. The IAS is modeled as sticks with zero radial diffusivity, as axons are much narrower than the diffusion length of the dMRI measurement. Within a macroscopic imaging voxel, elementary fascicles contribute to the directional dMRI signal according to their orientation distribution function (ODF) $P(\hat{n})$. (B) The Sensitivity-Specificity Matrix (SSM) $S_{ij}$ is defined in Eq. 8. An ideal SSM is an identity matrix. The diagonal elements measuring sensitivity are in bold for some SM estimator, the SSM is not square because the parameters that are not estimated (constrained) are left out. The nonzero off-diagonal elements reveal spurious correlations between model parameters, and are a hallmark of decreased specificity. The SSM is color-coded to highlight the elements that are greater in absolute values.

In the pursuit of specificity, there has been a growing interest (Novikov, Kiselev, et al., 2018) in biophysical models that directly parameterize relevant tissue microgeometry and thus offer ways to quantify its changes in health and disease (Alexander et al., 2019; Jelescu & Budde, 2017; Novikov et al., 2019). For WM, the *Standard Model* (SM) has been proposed as an overarching framework (Novikov, Veraart, et al., 2018; Novikov et al., 2019; Reisert et al., 2017), unifying multicompartment model-based strategies over the past two decades (Assaf et al., 2004; Kroenke et al., 2004; Jespersen et al., 2007; Alexander et al., 2010; Fieremans et al., 2011; Zhang et al., 2012; Kaden et al., 2016; Reisert et al., 2017; Novikov, Veraart, et al., 2018), see Figure 1A. In the SM, an elementary fiber fascicle comprises two non-exchanging compartments, the intra- and extra-axonal spaces (IAS and EAS). The SM offers an exciting potential of specificity to cellular-level biological phenomena, as its scalar parameters f, $D_{a}$, $D_{e}^{∥}$, $D_{e}^{⊥}$, as defined in Figure 1A and described in detail below, are by design more specific to micrometer-level manifestations of pathological processes.

In the intracellular space, the axonal water fraction (f) characterizes the relative contributions of IAS and EAS water. A decrease in f typically indicates axonal loss, suggesting a lower density of axons within the sampled voxel—a potential hallmark of neurodegeneration (Jelescu, Zurek, et al., 2016). A decrease in f may also indicate demyelination. The intra-axonal diffusivity ($D_{a}$) assesses axonal integrity. For instance, axonal injury, such as beading, disrupts the uniform diffusion pathway by introducing variations in axonal caliber, leading to the possible slowing or transient restriction of water molecules within axons (Budde & Frank, 2010; Lee, et al., 2020). Furthermore, within the EAS, the radial diffusivity ($D_{e}^{⊥}$) reflects the condition of the myelin sheath. The process of demyelination will reduce the complexity of pathways available to water molecules moving perpendicularly to the axons, resulting in increased $D_{e}^{⊥}$ (Jelescu, Zurek, et al., 2016). The axial diffusivity within the EAS ($D_{e}^{∥}$) detects a wider array of extra-axonal alterations. Decreases in $D_{e}^{∥}$ could indicate pathological events such as astrogliosis or microglial activation, both associated with neuroinflammatory responses (Xie et al., 2010).

The practical relevance of the SM is rooted in its assumptions which make it compatible with clinically feasible diffusion acquisitions as well as presence of a large number of publicly available dMRI datasets with multiple diffusion weightings $b=q^{2}t$ up to 2-3 $\text{ms}\;\;/\;\mu {\text{m}}^{2}$, such as UK Biobank (Miller et al., 2016), Human Connectome Project (Glasser et al., 2016), Alzheimer’s Disease Neuroimaging Initiative (Jack Jr et al., 2008), and Adolescent Brain Cognitive Development (Casey et al., 2018), together comprising hundreds of thousands of subjects. The application of the SM to these datasets presents unparalleled opportunities for large-scale *in vivo* studies of tissue microstructure in health and disease.

However, SM parameter estimation has proven to be challenging due to “shallow” (almost degenerate) directions in the likelihood landscape (Jelescu, Veraart, et al., 2016; Novikov, Veraart, et al., 2018). Conventional maximum likelihood estimation (MLE) suffers from low precision in this degenerate likelihood landscape. Recently, machine learning (ML)-based approaches have emerged as a promising tool for increased precision and speed. In the field of quantitative magnetic resonance imaging, ML approaches have been used to estimate ${\text{T}}_{1}$ and ${\text{T}}_{2}$ (Cohen et al., 2018), myelin water fraction (Liu et al., 2020), susceptibility (Yoon et al., 2018), and dMRI model parameters (Golkov et al., 2016; Palombo et al., 2020; Reisert et al., 2017).

Here, we quantify the sensitivity and specificity of ML-based SM estimation for relatively short (~6 min), clinically feasible dMRI protocols. Using dMRI acquired on patients during routine brain scans, we demonstrate how SM parameters are able to capture specific cellular-level changes in early development, stroke, and multiple sclerosis (MS). Our results open the way to apply this modern methodology in clinical settings and to large imaging consortium data (Casey et al., 2018; Glasser et al., 2016; Jack Jr et al., 2008; Miller et al., 2016) for investigating development, aging, and pathology.

## 2 Theory

### 2.1 Assumptions of the standard model

According to the SM, the dMRI signal originates from a collection of identical fiber fascicles in a WM voxel, that are oriented based on an arbitrary orientation density function (ODF) $P(\hat{n})$, Figure 1A. The following SM assumptions specify the physics of diffusion inside an elementary fascicle:

- First, the signal from a fascicle is a sum of contributions from non-exchanging spin populations in the IAS and EAS. Water exchange can be neglected since myelin layers form a practically impermeable boundary for the clinically feasible diffusion times.
- Second, the fascicle’s IAS is represented as a collection of aligned zero-radius cylinders (“sticks”), such that diffusion occurs only along the stick, while the transverse diffusion is negligible since axonal diameters of ~1 μm are much smaller than typical diffusion displacements in a clinical MRI measurement. The bulk along-stick diffusion coefficient $D_{a}$ is reduced relative to that of free water $D_{0}=3\;\mu \;{\text{m}}^{2}\;/\;\text{ms}$ due to intra-axonal organelles and micro-variations of axonal shape, such as beads (Lee et al., 2020). (Diffusivities and b-values are in the units of $\mu {\text{m}}^{2}\;/\;\text{ms}$ and $\text{ms}\;/\;\mu {\text{m}}^{2}$ throughout this work.)
- Third, diffusion in the fascicle’s EAS is assumed to be anisotropic and Gaussian, characterized by the axially symmetric tensor with parallel and perpendicular eigenvalues $D_{e}^{∥}$ and $D_{e}^{⊥}$. This means that diffusion at clinical diffusion times is assumed to be in the long-time limit, and any residual diffusion time-dependence (Novikov et al., 2014) is neglected.

This “impermeable stick” assumption has been verified *in vivo* for human WM by observing the distinct functional form at strong diffusion weightings that is inherent to such stick compartments (McKinnon et al., 2017; Veraart et al., 2019). The two SM compartments, IAS and EAS, define a response kernel for a fiber fascicle, which is a local bundle of aligned sticks with the EAS space surrounding them. The kernel’s signal is:

$$K\left(b,\xi \right)=s_{0}\;\left[fe^{-bD_{a}\xi^{2}}+\left(1-f\right)e^{-bD_{e}^{∥}\xi^{2}-bD_{e}^{⊥}\left(1-\xi^{2}\right)}\right]$$ (1)

where $\xi =\hat{g}\cdot \hat{n}$ is the scalar product between the symmetry axis $\hat{n}$ of the kernel and the gradient direction $\hat{g}$. Further compartments, such as isotropic cerebrospinal fluid (CSF), $\sim f_{iso}e^{-bD_{0}}$, can in principle be added. However, given typical multi-shell protocols with moderate b, the CSF fraction $f_{iso}$, which is a lot smaller than the intra-axonal water fraction f in WM, is very difficult to estimate. Figure S1 shows that multi-shell protocols are not sensitive enough to estimate $f_{iso}$ at realistic SNR. Introducing the free-water compartment will further increase the difficulty of estimating other SM parameters, especially EAS diffusivities (as the EAS signal is similar in its functional form to that of the CSF). Therefore, in this work, we use a two-compartment kernel (1) without CSF Liao, et al., 2024.

Such multicompartment fascicles are distributed in a voxel based on the fiber ODF. All fascicles are assumed to have the same compartment fractions and diffusivities, and differ from each other only by their orientation (Christiaens et al., 2020). Thus, the SM signal, measured along gradient direction $\hat{g}$, is a convolution between fiber response kernel $K(b,\hat{g}\cdot \hat{n})$ and the ODF $P(\hat{n})$ on a unit sphere:

$$S_{\hat{g}}(b)\int_{|\hat{n}|=1}d\hat{n}\;P(\hat{n})K(b,\hat{g}\cdot \hat{n})$$ (2)

where the ODF $P(\hat{n})$ is normalized to $\int_{|\hat{n}|=1}P(\hat{n})d\hat{n}=1$, and $\text{d}\hat{n}=\sin\theta \text{d}\theta \text{d}\phi /\;4\pi$ is the surface area element on a unit sphere normalized to unit area.

### 2.2 Rotational invariants in the spherical harmonic basis

We factorize the kernel from the ODF parameters in Eq. (2) using the spherical harmonic (SH) basis (Novikov, Veraart, et al., 2018; Reisert et al., 2017; Tournier et al., 2007):

$$S_{lm}\left(b\right)=p_{lm}K_{l}\left(b\right)$$ (3)

where $S_{lm}$ and $p_{lm}$ are the SH coefficients of the signal $S_{\hat{g}}\left(b\right)$ and of the ODF

$$P(\hat{n})\;\approx 1+\sum_{l=2,4,\ldots }^{l_{\max}}\sum_{m=-1}^{l}p_{\mathrm{lm}}Y_{\mathrm{lm}}(\hat{n})$$ (4)

up to order $l_{max}$ which practically depends on the dMRI sampling and signal-to-noise ratio (SNR). The functions $K_{l}\left(b\right)$ are projections of the kernel response onto the Legendre polynomials $P_{l}\left(\xi \right)$:

$$K_{l}\left(b\right)\equiv \int_{0}^{1}\text{d}\xi \;K\left(b,\xi \right)P_{l}\left(\xi \right)$$ (5)

To factor out the dependence on the choice of the physical basis in three-dimensional space (via m=−l…l), the rotational invariants are defined as follows (Novikov, Veraart, et al., 2018; Reisert et al., 2017):

$$\begin{array}{ll} S_{l}^{2}\left(b\right) & =\frac{1}{4\pi \left(2l+1\right)}\sum_{m=-l}^{l}|S_{lm}\left(b\right){|}^{2}\\ p_{l}^{2} & =\frac{1}{4\pi \left(2l+1\right)}\sum_{m=-l}^{l}|p_{lm}{|}^{2} \end{array}$$ (6)

From the relationship between rotational invariants and SH coefficients, one can relate the rotational invariants to the kernel parameters (Novikov, Veraart, et al., 2018; Reisert et al., 2017):

$$S_{l}\left(b\right)=p_{l}K_{l}\left(b\right),\;l=0,\;2,\;4,\;\ldots \;.$$ (7)

This enables a compression of raw directional dMRI measurements $S_{\hat{g}}\left(b\right)$ to a small number of data features $S_{l}$ without loss of information. Here, $p_{0}\equiv 1$ under the ODF normalization; the remaining ODF invariants, one for each l, characterize its anisotropy, with the normalization factor chosen so that $0<p_{l}<1$. Among these $p_{l},\;l=2,4,6...$, $p_{2}$ has the lowest order and thus highest SNR. Combining $p_{l}$ of the ODF with the kernel parameters, the SM parameters of interest are defined as $\theta =\left\{f,D_{a},D_{e}^{∥},D_{e}^{⊥};p_{2},p_{4},\ldots \right\}$. We will focus on $p_{2}$, as the most easily interpretable ODF alignment metric. While the SM also enables the deconvolution of the ODF SH coefficients based on voxelwise response kernel via Eq.(3), we will limit ourselves to the scalar metrics in this work.

### 2.3 Degeneracy of the estimation landscape

For any diffusion direction, the SM signal is a sum of decaying exponentials. Parameter estimation for models of such kind is generally ill-posed. Specific near-degenerate dimensions in the MLE landscape have been established for the SM numerically (Jelescu, Veraart, et al., 2016) and analytically (Novikov, Veraart, et al., 2018). In such a shallow MLE landscape, different combinations of model parameters may become indistinguishable in the presence of realistic noise, causing unstable estimation results.

To improve the robustness of estimation with limited dMRI data, conventional maximum likelihood estimators apply constraints. For instance, Neurite Orientation Dispersion and Density Imaging (NODDI) (Zhang et al., 2012) and Spherical Mean Technique (SMT) (Kaden et al., 2016) both assume $D_{a}\;=\;D_{e}^{∥}$, with NODDI further fixing $D_{a}$ and $D_{e}^{∥}$ to 1.7 $\mu {\text{m}}^{2}\;/\text{ms}$. Both NODDI and SMT use a tortuosity model to derive $D_{e}^{⊥}$: $D_{e}^{⊥}=D_{e}^{∥}\cdot \left(1-f\right)$ (Szafer et al., 1995). On the other hand, White Matter Tract Integrity (WMTI) (Fieremans et al., 2011) and Watson-WMTI (W-WMTI) (Jespersen et al., 2018) imply specific fiber ODF shapes and impose a square-root branch choice, $D_{a}\leq D_{e}^{∥}$ for WMTI and $D_{a}\;\geq D_{e}^{∥}$ for W-WMTI. Please refer to Table S1 for the conceptual comparison of these SM estimators, in particular their constraints. These overly simplified constraints may introduce biases and thus result in spurious findings (Jelescu et al., 2015; Jelescu, Veraart, et al., 2016; Lampinen et al., 2017; Novikov, Veraart, et al., 2018). Since NODDI fixes $D_{a}$ and $D_{e}^{∥}$ at 1.7 $\mu {\text{m}}^{2}\;/\text{ms}$, and SMT uses spherical average signals of b-shells to factor out ODF ($p_{l}$), their results may be left blank or omitted in the following figures when comparing the SM estimators.

### 2.4 How many independent scalar SM parameters can be estimated?

Fundamentally, the number of independent parameters one can in principle determine is tied to the information content of the dMRI signal (the number of independent features accessible from data at a given noise level). The estimation of the fascicle and ODF parameters factorizes in the spherical harmonics basis (Novikov, Veraart, et al., 2018; Reisert et al., 2017), Eq. (7), such that the number of independent scalar signal features $N_{b}N_{l}$ is a product of the number $N_{b}$ of the b-shells in the q-space, and the number $N_{l}$ of the independent *rotational invariants*$S_{l}\left(b\right)$ of the signal per shell (constructed from its spherical harmonics $S_{lm}\left(b\right)$, Eq. (6)) accessible at a given noise level.

In this work, as well as in publicly available dMRI datasets (Casey et al., 2018; Glasser et al., 2016; Jack Jr et al., 2008; Miller et al., 2016), all acquisitions have $N_{b}=2$ shells, and we use $S_{l}\left(b\right)$ with l=0,2,4, such that $N_{l}=3$, yielding overall 2×3=6 independent measurements for the fascicle response. This exactly matches the number of independent SM parameters $\theta =\left\{f,D_{a},D_{e}^{∥},D_{e}^{⊥};p_{2},p_{4}\right\}$ contributing at these l, Eq. (7). Note that employing the invariant $S_{4}\left(b\right)$ is crucial, since the system (7) with $S_{0}\left(b\right)$ and $S_{2}\left(b\right)$ for two b-shells yields only $N_{b}N_{l}=4$ independent measurements for five nonlinearly coupled parameters $\theta =\left\{f,D_{a},D_{e}^{∥},D_{e}^{⊥};p_{2}\right\}$. Practically, the signal invariants $S_{l}\left(b\right)$ decrease quickly with l; fortunately, maps of $S_{l}\left(b\right)$ up to l=4, as shown in Figure S2A, display clear WM structure. Figure S2B, C further demonstrates that $S_{4}\left(b\right)$ remains informative for the clinical applications discussed in this work.

### 2.5 Unconstrained parameter estimation with machine learning

As an alternative to MLE, an ML-based estimator was proposed to directly map rotational invariants $S_{l}\left(b\right)$ to the SM parameters $\theta_{i}$ (Reisert et al., 2017). The ML-based estimator uses a “soft” prior, as the prior distribution (training sets) implicitly regularizes the estimation in the training process, instead of imposing hard constraints on the model. Here, we use an extended version of this method, dubbed Standard Model Imaging (SMI) (Coelho et al., 2022), applicable to multidimensional dMRI. SMI uses third-order polynomial regressions to map $S_{l}\left(b\right)$ with l=0,2,4 to a set $\theta_{i}=\left\{f,D_{a},D_{e}^{∥},D_{e}^{⊥};p_{2},p_{4}\right\}$. The same Gaussian distribution of SM parameter set $\theta =\left\{f,D_{a},D_{e}^{∥},D_{e}^{⊥};p_{2}\right\}$ is used for training the ML-based estimator throughout this study with mean [0.5, 2, 2, 0.7, 0.45] and variance [0.06, 1, 1, 0.1, 0.06].

The five aforementioned estimators (SMI, NODDI, SMT, WMTI, and W-WMTI) effectively measure the same set of parameters under the SM framework, but adopt different constraints (as summarized in Table S1), therefore resulting in different outcomes and trends. It has become a crucial need to determine the most reliable (sensitive and specific) diffusion model estimator for routinely used multi-shell protocols, which will enable leveraging the enormous publicly available datasets. To evaluate the performance of SM estimators, below we propose a metric to quantify the sensitivity and specificity of parameter estimation, similar to the concept of the confusion matrix in classification. We then apply these estimators in various *in vivo* datasets, including early development, acute ischemia, and MS (total N=821), and compare them in light of the current knowledge of relevant (patho)physiological processes in the WM.

## 3 Methods and Materials

### 3.1 Subjects and dMRI acquisition

We studied various datasets that included two-shell dMRI scans ranging from 5 to 7 minutes long, that were acquired on patients referred for routine clinical brain MRI in the department of Radiology at New York University (NYU) and Medical University of South Carolina (MUSC), indicating the potential for clinical translation of the proposed methods. Institutional Internal Review Board approval with waiver of consent was obtained for retrospective study.

#### 3.1.1 Early development

For the assessment of human development, brain MRIs from 59 pediatric subjects (30 females) who underwent DKI imaging as part of a routine MRI exam under sedation at NYU School of Medicine from June 2009 to October 2010 were analyzed (Jelescu et al., 2015; Paydar et al., 2014). The subjects ranged from 1 day to 2 years and 9 months in age, and all underwent brain MR imaging for non-neurological indications. All the included exams were interpreted as normal by fellowship-trained board-certified neuroradiologists, and were reevaluated by a board-certified pediatric neuroradiologist for normalcy prior to inclusion.

All pediatric subjects were scanned on a 1.5 T Siemens Avanto MRI scanner using a body coil for excitation and an 8-channel head coil for reception (Jelescu et al., 2015; Paydar et al., 2014). Whole-brain diffusion weighted data were acquired using twice-refocused spin-echo, single shot echo planar imaging with 1 b = 0 image and along 30 diffusion encoding directions for b = 1, 2 $\text{ms}\;/\;\mu {\text{m}}^{2}$. Other parameters included: TR/TE: 4500/96 ms, matrix size: 82 × 82; 28–34 slices (no gap); and voxel size of 2.2–2.7 × 2.2–2.7 × 4–5 ${\text{mm}}^{3}$, 1 average, acquisition time approximately 5 minutes.

#### 3.1.2 Ischemia

For the assessment of (sub)acute stroke (Hui et al., 2012), clinical and MRI data were reviewed for consecutive patients who were admitted to MUSC with acute onset of neurological symptoms. These patients were subsequently diagnosed with acute or subacute stroke in the middle cerebral artery territory as the cause of their neurological impairments. A total of 28 patients admitted to this institution between August 2011 and February 2012 were included. These patients underwent MRI 7 hours to 3 weeks after symptom onset (82% of the stroke patients were scanned within the first week of symptom onset). Patients with a history of brain neoplasm or intracranial hemorrhages were excluded from study.

The stroke patients were scanned on a 1.5 T Siemens Avanto MRI scanner (Hui et al., 2012). Diffusion-weighted images were acquired with 3 b-values (1 b = 0 image; 1 and 2 $\text{ms}\;/\;\mu {\text{m}}^{2}$ along 30 diffusion encoding directions) using a vendor-supplied single-shot twice-refocused spin-echo echoplanar imaging sequence. Other imaging parameters were: slice thickness = 3 mm (no gap), number of slices = 40, TR/TE = 5500/99 ms, field-of-view = 222 × 222 ${\text{mm}}^{2}$, acquisition matrix = 74 × 74, image resolution = 3 × 3 ${\text{mm}}^{2}$, acceleration factor 2, and acquisition time approximately 7 minutes.

#### 3.1.3 Multiple sclerosis and healthy controls

For the assessment of MS, we studied 177 subjects (age 48.47 ± 9.78 years old, 119 females) identified with a clinical diagnosis of MS using the McDonald criteria (Polman et al., 2011) who were referred for MRI of the head at NYU Langone Health between November 2014 and June 2020. Within 1 year of the MRI, disability status was assessed using the Patient Determined Disease Steps (PDDS) questionnaire, a validated nine-point patient-reported metric of disease severity (Kister et al., 2013). MS patients were separated into mild MS (0,,≤PDDS≤3) and severe MS (4≤PDDS≤7) based on the need of canes for walking.

In total, 557 healthy controls (age 45.29 ± 13.94 years old, 388 females) were selected from patients with normal brain MRI, and no history of neurological disorder. The subjects were referred for MRI of the head at NYU Langone Health due to headache or dizziness. To compare with the MS patients, 177 of healthy subjects (age 48.47 ± 9.76 years old, 119 females) matched by age and sex were chosen as controls. Moreover, 177 adults aged between 25 and 35 years old (age 30.28 ± 2.91 years old, 94 females) were selected out of the cohort to establish the normative values of SM parameters to compare with the pediatric population.

Both MS patients and controls underwent clinically indicated MRI on a 3 T Siemens Magnetom Prisma (46.3%) or Skyra (53.7%) scanner. The dMRI protocol included a monopolar EPI sequence as follows: 4–5 b = 0 images, b = 1 $\text{ms}\;/\;\mu {\text{m}}^{2}$ along 20 directions and b = 2 $\text{ms}\;/\;\mu {\text{m}}^{2}$ along 60 directions, with imaging parameters: 50 slices, 130 × 130 matrix, voxel size = 1.7 × 1.7 × 3 mm, TE = 70–96 ms and TR = 3200–4000 ms on Prisma, TE = 95–100 ms and TR = 3500–4300 ms on Skyra, GRAPPA acceleration 2, and multiband 2. The total acquisition time was approximately 6 minutes.

### 3.2 dMRI processing

The dMRI data were processed using DESIGNER (Ades-Aron et al., 2018; Chen, et al., (2023)) for denoising (Veraart et al., 2016), Gibbs artifact correction (Lee et al., 2021), EPI-induced distortion correction (Andersson et al., 2003), motion and eddy current artifact correction (Smith et al., 2004), and Rician noise floor correction (Koay et al., 2009). Regions of interest (ROI) were automatically segmented by a nonlinear mapping onto the WM label atlas of Johns Hopkins University (JHU) (Mori et al., 2005). To mitigate the partial volume effects, we shrank each WM region by 1 voxel relative to the 1 mm template of the JHU atlas. The genu of corpus callosum (GCC) was chosen as a representative ROI for MS studies because it is a large homogeneous region in the corpus callosum with relatively few outliers. MS lesions, identified using icometrix (Rakić et al., 2021), are notably heterogeneous (Rovaris et al., 2005) with potential exchange between the IAS and EAS in the case of unmyelinated axons and possibly additional compartments due to increased inflammation. Characterizing MS lesions was beyond the scope of this study. Hence, we focused on comparing the normal appearing white matter (NAWM) in the MS subjects with that of healthy controls. For the stroke patients, the WM mask was determined by fractional anisotropy greater than 0.2 to include more voxels to the ROI for small ischemic lesions.

SM parameters were estimated using the five WM estimators described, which are SMI, NODDI, SMT, WMTI, and W-WMTI. The mean of an ROI was extracted for further analysis after excluding outliers. The voxels with unphysical SM parameter values were first excluded, then parameter values ±2σ away from the ROI mean were considered outliers, where σ is the standard deviation within an ROI. Typically, fewer than 5% of the voxels were discarded.

### 3.3 Statistical analysis

An exponential function (A⋅exp(−t/τ)+B) was fitted to the dataset of early development. The absolute value of τ was used to quantify the pace of the exponential growth or decay. For the stroke patients, the mean of ischemic lesions and their contralateral hemisphere in the WM were compared pairwise. The relative changes of ischemic lesions from their contralateral regions were calculated to quantify the degree of change for each SM parameter. In the MS study, MS patients were separated into two groups: mild MS patients and severe MS patients based on the PDDS score. ANCOVA was used to study the group differences between every two groups covarying for age.

### 3.4 Sensitivity-specificity matrix

To quantify sensitivity and spurious correlations of parameter estimation, we considered the *Sensitivity-Specificity Matrix* (SSM) in noise propagations

$$S_{ij}=\frac{\langle \theta_{i}\rangle }{\langle \theta_{j}\rangle }\langle \frac{\partial {\hat{\theta }}_{j}}{\partial \theta_{i}}\rangle ,\;i,j=1,\ldots ,N_{\theta }$$ (8)

whose elements quantify relative changes of an estimated parameter ${\hat{\theta }}_{j}$ with respect to the actual change of parameter $\theta_{i}$. Here, the angular brackets denote averaging over the distribution of ground truths (the test set) of all $N_{\theta }$ parameters. The normalization by the mean values $\langle \theta_{i}\rangle$ was introduced for convenience, to make the off-diagonal elements dimensionless (and are redundant for the diagonal elements). Practically, we evaluated the SSM from a linear regression of the estimates ${\hat{\theta }}_{j}$ with respect to ground truths $\theta_{i}$ of all $N_{\theta }$ parameters in a test set.

Likewise, a linear regression of the estimated parameters was applied to the prior mean of SMI to demonstrate the dependency of ML-based estimation on the prior. We defined a matrix quantifying such impact as

$$P_{ij}=\frac{\langle \theta_{i}\rangle }{\langle \theta_{j}\rangle }\langle \frac{\partial {\hat{\theta }}_{j}}{\partial \mu_{\theta_{i}}}\rangle ,$$ (9)

where $\mu_{\theta_{i}}$ are the mean values of the prior distribution for each model parameter. While fixing the variance of the prior distribution of SM parameters at [0.06, 1, 1, 0.1, 0.06], the prior mean was varied from 90% to 110% of the reference [0.5, 2, 2, 0.7, 0.45] at the step of 2.5% for each parameter separately.

The synthetic data were generated based on the two-compartment SM using a two-shell protocol (same as *in vivo* controls). The ODF was simulated by spherical harmonics up to l=4. The ratio of $p_{4}$ to $p_{2}$ was set between 0.75 and 0.85 according to histology results (Lee et al., 2019). Gaussian noise was added to the signal at SNR = 25 with respect to ${\text{b}}_{0}$ images. To evaluate the SSM, the ground truth of SM parameters $\theta =\left\{f,D_{a},D_{e}^{∥},D_{e}^{⊥};p_{2}\right\}$ was uniformly sampled 10,000 times from [0.3, 1.5, 1.5, 0.4, 0.3] and [0.8, 2.5, 2.5, 1, 0.8] to focus on the most probable parameter range in WM voxels. Note that $D_{e}^{∥}\geq D_{e}^{⊥}$ was enforced in our simulation given their definition.

## 4 Results and Discussion

### 4.1 Sensitivity and specificity of SM parameters in simulations

Results in Figure 1B show that SMI provides the most trustworthy estimates of SM parameters. In particular, it estimates $p_{2}$, f and $D_{a}$ almost free of spurious correlations. On the other hand, the SSM (Fig. 1B) reveals that SM parameters estimated by NODDI have spurious correlations with one another, notably SSM $(D_{a},{\hat{p}}_{2})=0.51$ and SSM $(D_{a},\hat{\;f})=-0.48$. These spurious correlations are particularly concerning in the case of a significant change in $D_{a}$, for example, in pathology, that would translate into apparent changes of f and $p_{2}$. The estimation of $D_{a}$ by SMT has a combination of contrasts from $D_{a}$, $D_{e}^{∥}$ and f, and the estimation of $D_{a}$ by WMTI has a combination of contrasts of $D_{a}$ and $p_{2}$. These spurious correlations revealed by the SSM are caused by limited information obtainable from multi-shell dMRI scans and imposing hard constraints on the SM.

We show in Figure S3 the relationship between SM estimates and the prior mean given the same variance, which suggests the lower the sensitivity and specificity are, the more strongly the estimates are influenced by the prior distribution (training set). Their relationship is close to linear at the realistic SNR of dMRI (Fig. S3). Furthermore, the scatter plots of estimated parameters against ground truth for numerical noise propagations are shown in Figure S4A for all five estimators.

Specificity of the SM parameters has also been validated through histological studies. Coronado-Leija et al. (2023) recently performed, in a rat model of chronic traumatic brain injury, an extensive comparison of dMRI-derived SM parameters against histology, evaluating different models, including SMI and NODDI. They have demonstrated that the SM parameters for fiber dispersion are in excellent agreement with those derived from 3 d electron microscopic images. Furthermore, the intra-axonal diffusivity agrees with the estimate from histology (based on the variation in the axon diameter). This work provides robust validation for SM parameters and demonstrates their specificity to geometric microscopic properties of WM tissues.

SM parameters have been demonstrated to be sensitive to various WM pathological processes. Notably, Jelescu, Zurek, et al. (2016) have shown that demyelination leads to a decrease in f and an increase in $D_{e}^{⊥}$, in a mouse model of de- and remyelination, suggesting there is no one-to-one correspondence between these two SM parameters, and both myelination and demyelination can influence f and $D_{e}^{⊥}$ simultaneously. On the other hand, pruning will predominantly reduce the anisotropy of axon fibers ($p_{2}$) and to a lesser extent result in axonal loss (f). To maintain clarity in interpretation of the following SM findings, we focus on discussing the primary effects of specific processes.

### 4.2 Disentanglement of sequential processes in early development

**Fig. 2.**
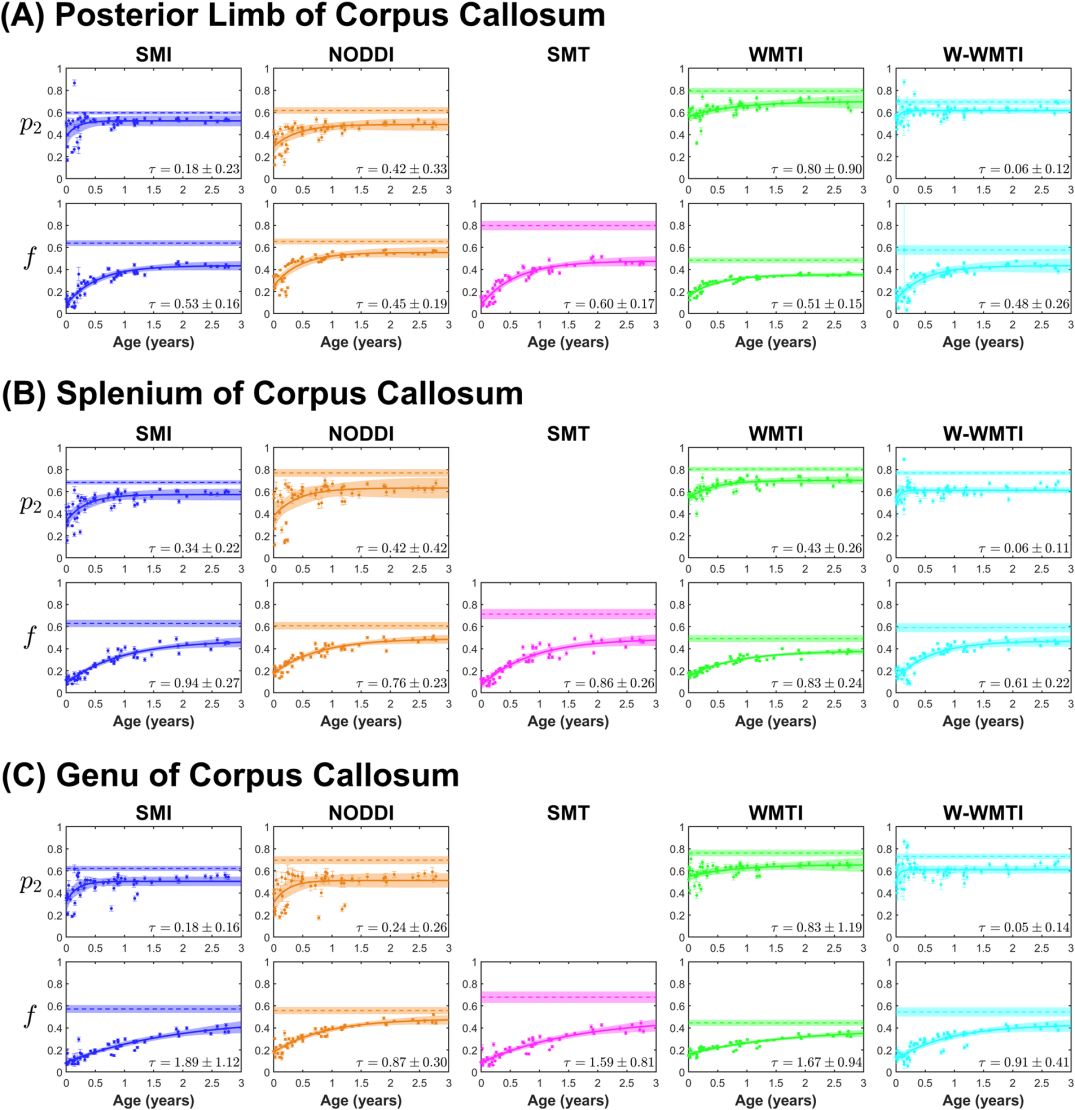
Early development trends of diffusion parameters in white matter regions. SM parameters have distinct paces during early development, likely reflecting different developmental processes. Early development trends are shown for (A) posterior limb of corpus callosum, (B) splenium of corpus callosum, and (C) genu of corpus callosum, presented in the order of their maturation. Data points in each plot represent the ROI mean values for the pediatric subjects, and error bars indicate their 95% confidence interval. The exponential fit of the development data is plotted as a solid line within the 95% confidence interval, while the 95% confidence interval of its time constant τ is indicated on each plot. As a reference for the pediatric data, the dashed line and its neighboring shaded area represent the mean and standard deviation of the corresponding ROI mean from 177 controls aged between 25 and 35 years old.

Pediatric subjects (N = 59) aged between 0 and 3 years old were selected for the study of early development (Jelescu et al., 2015; Paydar et al., 2014). As a reference for the pediatric data, 177 healthy controls aged between 25 and 35 years old were also selected. More detailed parameter distribution for healthy controls in the WM are presented in Figure S4B. Developmental trajectories of $p_{2}$ and f are illustrated in Figure 2 for three WM regions. These two parameters were selected for detailed investigation because they are the most robust parameters of all SM parameters according to simulation results in Figure 1B.

The results by NODDI for newborns align with previous studies using NODDI (Dean III et al., 2017; Kunz et al., 2014). DiPiero et al. (2023) offer a comprehensive review of dMRI studies focusing on early development. The overall trends of early human brain development are largely consistent across the five estimators for $p_{2}$ and f. Yet, they differ in the pace of developmental processes, which can be quantified by the time constant τ of an exponential functional form A exp(−t/τ)+B for its dynamics.

In human brains, axonal and synaptic pruning of human brains occur in two significant phases: the first 2 years after birth and during adolescence, with the latter more activity-dependent (Riccomagno & Kolodkin, 2015). During the first 2 years, new synapses are also rapidly formed through synaptogenesis in the human cerebral cortex, with synaptic density peaking at 1 to 2 years of age, approximately 50% above adult levels (Huttenlocher et al., 1979). In the context of deep white matter, axonal pruning is more pertinent to our study. An exuberant network of neural connections is initially generated and later remodeled by a wide variety of cellular strategies (Cowan et al., 1984; Innocenti & Price, 2005; Luo & O’Leary, 2005). During development, neurons typically extend axons to more targets than necessary for normal adult functioning. Both the fine-tuning of short axonal arbors and the elimination of long axon collaterals are crucial aspects of developmental axonal pruning (Luo & O’Leary, 2005). Evidence from post-mortem animal studies demonstrates a significant reduction of axons in the pyramidal tracts of cats (Berbel & Innocenti, 1988), corpus callosum of rats (Gorgels, 1990), and rhesus monkeys (LaMantia & Rakic, 1990) shortly after birth and consistently preceding myelination. Axonal pruning likely removes redundant neural connections, while the remaining relevant circuits are strengthened through ongoing axonal growth (Faust et al., 2021), contributing to increased fiber anisotropy.

Based on the SSM in Figure 1B, $p_{2}$ of SMI is the most robust parameter among all SM parameters and shows minimal spurious correlations with SM kernel parameters. NODDI also demonstrates fairly high accuracy in estimating $p_{2}$ according to the simulation results (Fig. 1B) despite some spurious correlations with the compartmental axial diffusivities, which appear to be stable in early development (Fig. S5). Both SMI and NODDI, the two SM estimators that most accurately measure $p_{2}$, detect an increase of fiber anisotropy within the first few months after birth ($\tau (p_{2})<0.4$ year for all WM regions in Table S2). Furthermore, Table S2 shows that $\tau (p_{2})$-values are consistently smaller than τ(f)-values across all WM regions. This trend suggests that $p_{2}$ likely captures a developmental process that proceeds faster than myelination and axonal growth, which are the primary drivers of the change in f. The time constants of $p_{2}$ are in line with the time scale of axonal pruning shown in post-mortem animal studies (Berbel & Innocenti, 1988; Gorgels, 1990; LaMantia & Rakic, 1990).

Both axon diameter growth and myelination span extensively, persisting into adulthood (Miller et al., 2012). The “impermeable stick” assumption of the SM might not hold for axons that are not yet myelinated. Thus, the markedly low axonal water fraction at birth likely indicates the fraction of only the myelinated axons and not the unmyelinated axons. This suggests that unmyelinated axons may be permeable for the diffusion times employed in clinical dMRI (estimated to be around 50 ms based on TE = 96 ms), whereas the myelinated axons are impermeable at this time scale. Therefore, f is more indicative of myelination. The time constants of f shown in Table S2 are generally consistent with the 1 ~2 years of time constant measured from myelin water fraction (Deoni et al., 2012).

Furthermore, regional variation of diffusion in development has been explored in various studies using DTI (Lebel et al., 2008), DKI (Paydar et al., 2014), WMTI, and NODDI (Jelescu et al., 2015). Their findings are generally consistent with the estimations of SMI (Table S2) and follow the expected regional variation. In particular, this is illustrated in Figure 2 for the intra-axonal fraction (f) estimated by SMI, resulting in following time constants (years): posterior limb of internal capsule (0.53±0.16), splenium of corpus callosum (0.94±0.27), and genu of corpus callosum (1.89±1.12). These early development trends in individual WM regions conform to the neuroscience principle that WM matures in a posterior-to-anterior and inferior-to-superior manner (Colby et al., 2011; Yakovlev, 1967).

Values for $D_{a}$, $D_{e}^{∥}$ and $D_{e}^{⊥}$ in early development are plotted in Figure S5. The axial compartmental diffusivities are remarkably stable over the course of early development. Potentially higher fluid content in early development would result in a substantial overestimation in $D_{e}^{∥}$ by SMI, which is not observed in our data. In addition, $D_{e}^{⊥}$ demonstrates a decline over the course of first 3 years after birth, consistent with the process of myelination hindering radial diffusion in the EAS.

### 4.3 Detection of axonal beading in ischemic lesions

**Fig. 3.**
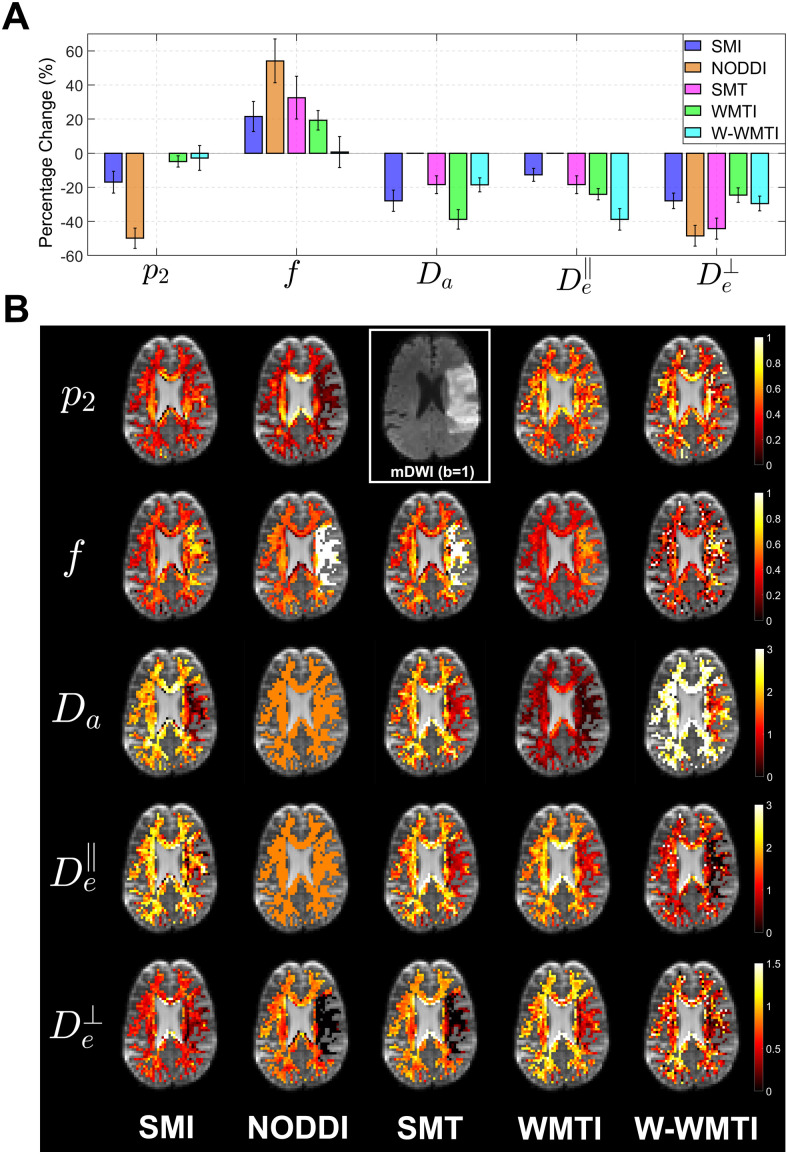
White matter microstructure parameter changes in ischemic lesions as compared to the contralateral hemisphere. (A) Mean relative percent changes in SM parameters for 28 subjects, comparing normal tissue (in the contralateral hemisphere) to (sub)acute ischemic tissues, are shown in the bar plot, with error bars representing the 95% confidence interval. (B) Parametric maps of WM are overlaid on the b = 0 images of an exemplary stroke patient (scanned 26 hours after the onset of ischemia). In the middle of the top row (within the white box) is the mean DWI (mDWI) signal averaged over different directions of the $b=1\;\mu {\text{m}}^{2}\;/\text{ms}$ shell, where the ischemic lesion is clearly shown.

Subjects (N = 28) suffering from stroke and imaged with MRI from 6 hours to 2 weeks after ischemic onset were selected for the study of (sub)acute ischemia (Hui et al., 2012). Figure 3A shows the relative change of SM parameters in ischemic WM lesions compared to the same regions in the contralateral hemisphere. It is exemplified by the parametric maps of a stroke patient scanned 26 hours after the onset of ischemia in Figure 3B. Remarkably, the $D_{a}$ map of SMI reveals that $D_{a}$ drops below 0.5 $\mu {\text{m}}^{2}\;/\;\text{ms}$ in a large portion of the ischemic region while it is around 1.5 $\mu {\text{m}}^{2}/\text{ms}$ in the contralateral hemisphere. In line with the parametric maps, the bar plots of SMI show $D_{a}$ and $D_{e}^{⊥}$ experience the largest decrease of ~30% averaged over all stroke patients, while f increases by ~20% and $D_{e}^{∥}$ decreases by ~15%.

Cytotoxic edema, commonly observed after a stroke, arises from the movement of water from the interstitial space into cells (Heo et al., 2005). This phenomenon occurs when extracellular Na^+^ and other cations accumulate inside neurons and astrocytes, partly due to the breakdown of energy-dependent extrusion mechanisms (Liang et al., 2007). dMRI is acutely sensitive to cerebral ischemia; the ADC from a dMRI scan drops dramatically within the infarcted region shortly after an ischemic event. From a microstructure perspective, Budde and Frank (2010) proposed that axonal beading could lead to a significant reduction in ADC, a hypothesis supported by simulations and in vitro experiments. Furthermore, Novikov et al. (2014) demonstrated that the structural disorder in the brain post-stroke is one-dimensional, based on diffusion time-dependence, a finding that corroborates the axonal beading model. In this context of axonal beading and cytotoxic edema, we anticipate the most profound change in intra-axonal diffusion being reduced due to beading ($D_{a}$), to a lesser extent an increase in axonal space (f) from the beading, and a reduction in the extra-cellular diffusion, axially ($D_{e}^{∥}$) and radially ($D_{e}^{⊥}$), due to extracellular constriction. SMI results are consistent with anticipated changes from axonal beading and cytotoxic edema. On the other hand, NODDI and SMT produce unphysical f and $p_{2}$ values, indicating the assumptions $D_{a}=D_{e}^{∥}=1.7\;\mu {\text{m}}^{2}\;/\;\text{ms}$ or $D_{a}=D_{e}^{∥}$ might not apply during ischemia.

### 4.4 Detection of axonal loss and demyelination in multiple sclerosis

**Fig. 4.**
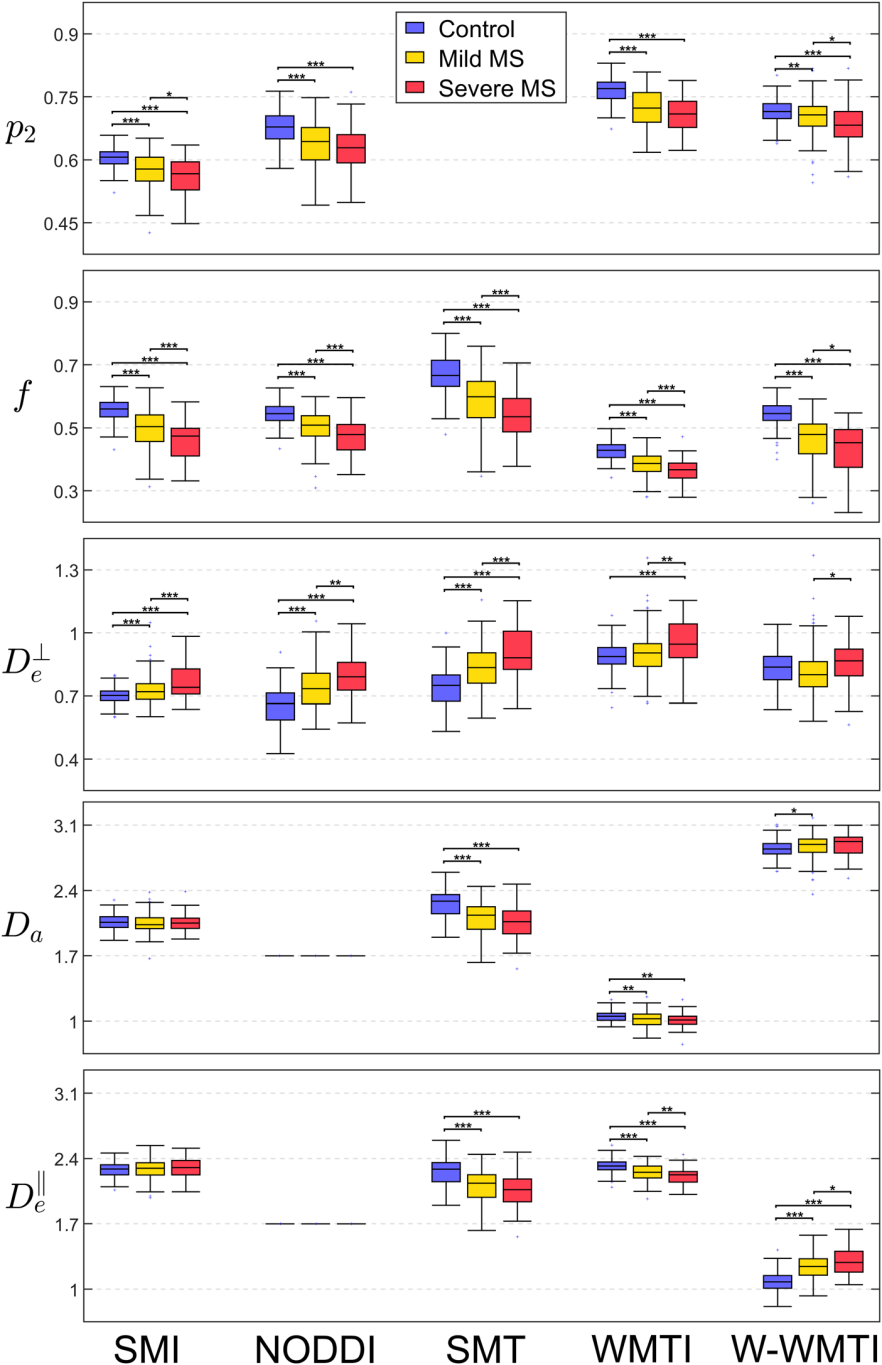
Box plots of white matter microstructure parameters in MS patients and controls. In comparison to controls (blue), MS patients are separated into mild MS (yellow) and severe MS (red). The mean of GCC is shown in the box plot after correcting for age. Please note that all diffusivities estimated by NODDI are constrained, i.e., $D_{a}=D_{e}^{∥}=1.7\;\mu {\text{ m}}^{2}\;/\text{ms}$, $D_{e}^{⊥}=D_{e}^{∥}\cdot \left(1-f\right)$, while SMT assumes $D_{a}$ and $D_{e}^{∥}$ are identical but not fixed. In each box, the central mark indicates the median, and the bottom and top edges of the box indicate the 25th and 75th percentiles, respectively. The whiskers extend to the most extreme data points not considered outliers, and the outliers are plotted individually using the “+” marker symbol. The significance levels of the statistical ANCOVA test are displayed in asterisks on top of every two groups (*: p < .05; **: p < .01; ***: p < .001).

MS patients (N = 177, 134 mild, 43 severe) were compared with 177 age and sex-matched controls in the GCC. MS lesions have been masked out from the GCC before comparison. The severity of MS is determined based on the PDDS questionnaire (mild: 0≤ PDDS ≤3, severe: 4≤ PDDS ≤7) (Kister et al., 2013), where the clinical distinction between mild and severe MS is determined based on the ability to walk without a cane. The mean values of GCC normalized for age are shown in Figure 4.

MS patients show lower f and higher $D_{e}^{⊥}$ compared to controls, which is consistent with known MS pathology of axonal loss and demyelination (Trapp & Nave, 2008). The decrease of $p_{2}$ in MS patients could be induced by the activation of microglia in the neuroinflammatory response (Voet et al., 2019). An increasing number of microglia, which are morphologically plastic and considerably larger in size than axon diameters (Lawson et al., 1990), may reduce the apparent anisotropy of ODF. While the five estimators detect largely consistent changes between controls and MS patients in $p_{2}$, f and $D_{e}^{⊥}$, SMI is the only estimator that captures the continuous trend of MS severity from mild to severe cases in all three parameters. Nevertheless, the changes in $D_{a}$ and $D_{e}^{∥}$ are inconsistent among the five estimators (discussed below in section 4.5).

### 4.5 Source of discrepancies between SM estimators explained by the sensitivity-specificity matrix

Cellular and pathological specificity is the primary motivation for biophysical modeling of dMRI signals in brain WM. Yet, due to the limited information available in multi-shell dMRI protocols, biophysical models commonly employ model constraints to stabilize parameter estimation. These constraints tend to introduce unknown systematic biases and lead to discrepancies in parameter quantification and group comparisons. Despite efforts to compare the results of different WM estimators for the same dataset (Beck et al., 2021; Jelescu et al., 2015), the source of discrepancies so far has not been fully explained. We address this gap by proposing the SSM as a means to quantify the sensitivity and specificity of parameter estimation and thereby provide an explanation for the source of discrepancies in different estimators. The usefulness of SSM is illustrated here in various clinical datasets.

In early development, the SSM suggests SMI has the highest specificity in estimating $p_{2}$ and f. Indeed, SMI shows that the increase of $p_{2}$ is consistently faster than that of f across all WM regions (Table S2). The two parameters are clearly distinguished by the pace of their growth.

In ischemic lesions, the SSM predicts that SMI can capture the drop in $D_{a}$, while both SMT, and to an even larger extent NODDI, will instead cause the estimated $\hat{\;f}$ to increase (Fig. 3), since NODDI completely fixes $D_{a}$ and SMT still allows $D_{a}$ to be estimated.

In MS, the primary pathological processes, axonal loss, demyelination, and inflammation, affect f, $D_{e}^{⊥}$ and $p_{2}$ the most, whereas $D_{a}$ and $D_{e}^{∥}$ are less impacted. According to the results of SMI, there is no significant change of $D_{a}$ and $D_{e}^{∥}$ detected between controls and MS patients. Yet, the changes of f, $p_{2}$ and $D_{e}^{⊥}$ may “leak” into the estimation of $D_{a}$ and $D_{e}^{∥}$. For instance, ${\hat{D}}_{a}$ estimated by SMT has a positive relationship with f (SSM $(f,{\hat{D}}_{a})=0.37$), which leads to the finding of a significant decrease in $D_{a}$. Likewise, ${\hat{D}}_{a}$ estimated by WMTI has a positive relationship with $p_{2}\;\;(SSM\;\;(p_{2},\;\;{\hat{D}}_{a})=0.86)$, which may explain the significant decrease of $D_{a}$ detected by WMTI. The same rationale can be applied to further explain the other discrepancies in the MS results of $D_{a}$ and $D_{e}^{∥}$.

### 4.6 ML-based estimator vs maximum likelihood estimation

For the multi-shell dMRI protocols employed in clinical and large-scale studies (Casey et al., 2018; Glasser et al., 2016; Jack Jr et al., 2008; Miller et al., 2016), the limited information necessitates employing constraints to stabilize MLE in a “shallow” (almost degenerate) optimization landscape (Jelescu, Veraart, et al., 2016; Novikov, Veraart, et al., 2018). Constraints like $D_{a}=D_{e}^{∥}$ in SMT, or further $D_{a}=D_{e}^{∥}=1.7$$\mu {\text{m}}^{2}\;/\text{ms}$ in NODDI significantly narrow down the shallow likelihood landscape and increase precision for f and $p_{2}$, which is why these constrained estimators have been embraced. However, such constrained estimators introduce biases to the estimation and result in spurious findings (Jelescu et al., 2015; Jelescu, Veraart, et al., 2016; Lampinen et al., 2017; Novikov, Veraart, et al., 2018). WMTI takes a different approach by relating SM parameters to DKI metrics. To establish this analytically, WMTI assumes fiber alignment, essentially forcing $p_{2}$ close to 1. It is no surprise to see all of parameters estimated by WMTI are correlated with $p_{2}$ (Fig. 1B). However, all SM parameters are not fixed throughout development, aging, and pathology. Hence, while an SM parameter can be fixed during the estimation process, the influence of a parameter may be reflected as changes in the other parameters, leading to spurious correlations.

ML-based methods provide a promising alternative and hold several unique advantages over MLE, which usually relies on applying hard constraints to achieve robustness. First, they optimize the training error of all samples as a whole rather than one sample at a time as in MLE, which allows the ML-based estimator to learn the trade-off between bias and variance. Second, ML-based estimators can learn an individual mapping from signals to every parameter, whereas MLE can only search for the most probable combination of parameters. The learning approach enhances the sensitivity of estimators to parameters that are confounded by others. Third, ML-based estimators use a “soft” prior distribution as training sets to regularize the estimation instead of applying hard constraints. The estimation of $p_{2}$ or f by SMI does not depend on the diffusivity as evidenced by the lack of spurious correlations between them. This is because the ML model learns to estimate $p_{2}$ and f through the training samples with varying diffusivities and is able to resolve them. In comparison, imposing hard constraints on diffusivities like in NODDI and SMT to stabilize the estimation process will inevitably lead to spurious correlations between the estimated f and the ground truth of diffusivities, as shown in their SSM (Fig. 1B). Fourth, SMI relies on rotational invariants up to $S_{4}\left(b\right)$, which is more informative than, say, SMT relying only on $S_{0}\left(b\right)$.

### 4.7 Limitations of the Standard Model

By releasing hard constraints and using a “soft” prior in the training, SMI achieves higher sensitivity and specificity than conventional MLE algorithms. However, there are still several caveats. First, despite a large number of datasets available, only very limited information regarding the tissue microstructure of WM can be obtained from multi-shell protocols, which results in the spurious correlations revealed by the SSM. To further improve the parameter estimation of the SM, incorporating extra “orthogonal” measurements is key in extracting complementary information about the tissue microstructure for the ML algorithms to learn from. Planar and spherical diffusion encodings, and measuring dMRI signals at multiple echo times have been shown to significantly improve the estimation precision and accuracy (Coelho et al., 2019, 2022; Lampinen et al., 2020; Reisert et al., 2019; Topgaard, 2017; Veraart et al., 2018).

Second, the SM may not be fully valid during early developmental stages or under certain pathological conditions. During very early development, there is minimal myelin around axons, potentially leading to water exchange between the IAS and EAS at the diffusion times employed in clinical diffusion MR protocols. We hypothesize that the SM parameter estimates might only measure myelinated axonal volume fractions when applied to developing WM. This hypothesis gains support from observations made from 0 to 3 years of age. During this period, the intra-axonal fraction f starts from notably low values and gradually approaches adult levels (Fig. 2). The time constants of f are largely consistent with results of myelin water imaging (Deoni et al., 2012).

Third, since SMI is an ML-based estimator, it is inherently influenced by the choice of training sets. Figure S3 illustrates that alterations to the prior mean induce a shift in the overall distribution. The amount of shift induced by the prior distribution for a given parameter is the complement of its sensitivity (diagonal elements of the SSM). Thus, the impact of the prior distribution is more pronounced for parameters that are more difficult to estimate, such as compartmental diffusivities. As a result, the absolute values of estimates by SMI are only comparable using the same prior distribution. The ML-based estimator aims to minimize the mean squared error, which is a composite of both bias and variance. In this minimization process, a reduction in variance is attained at the expense of increased bias. This dynamic essentially nudges the estimates closer to the prior mean. The bias introduced in this manner is systematic, meaning it will not affect the outcome in group comparisons. To better control the prior mean, we use a Gaussian prior centered at a predefined value, with a cutoff at the physical bounds. The variance of the Gaussian prior mediates the bias-variance tradeoff inherent to the estimation process. Lowering the variance of prior distributions enhances the precision of estimates located at the center of the prior distribution, albeit potentially increasing the bias of estimates at the outer range of the prior.

It is crucial to understand that the bias observed for the ML-based estimation is not inherently a product of the prior itself. Instead, it emerges due to the insufficient information provided by the acquisition protocol and SNR, which are imperative for accurately resolving the parameters. The prior distributions adopted in this study do cover the entire physical range and are general enough to process various datasets. The mean of the prior distribution is substantiated by results from an extensive protocol with maximum b-value up to 10 $\text{ms}\;/\;\mu {\text{m}}^{2}$ (Novikov, Veraart, et al., 2018). The variance of the prior distribution is selected to optimize the SSM for the multi-shell dMRI protocol at realistic SNR. This prior is recommended to be used in future studies with multi-shell dMRI protocols for comparison purposes.

## 5 Conclusion

In conclusion, because of the unique advantages enabled by ML-based estimators, as well as by relying upon the complementary rotational invariants $S_{l}\left(b\right)$ with l=0,2,4, SMI captures the biologically sensible morphological trends across three different clinical datasets, including early development, acute ischemia, and MS. The SSM was proposed to measure the sensitivity and specificity of parameter estimation, which largely explains the source of discrepancies between SM estimators. The SSM also demonstrates that SMI has the highest sensitivity and specificity among the five SM estimators in this study. Hence, SMI can serve as a powerful tool in clinical settings and for large imaging consortium data to study WM microstructure in a wide range of neurological diseases.

## Supplementary Material

Supplementary Material

## Data Availability

The code for the ML-based estimation is now publicly available in the SMI toolbox (https://github.com/NYU-DiffusionMRI/SMI). The code for NODDI, SMT, WMTI, and Watson-WMTI has been made public already. Data are available upon reasonable request from a qualified investigator and use agreement with the corresponding author.
