# Supplementary material for: Mapping tissue microstructure of brain white matter in vivo in health and disease using diffusion MRI
Source: Imaging Neurosci (Camb). 2024 Mar 6;2:imag-2-00102. doi: 10.1162/imag_a_00102 (PMC12224470; doi:10.1162/imag_a_00102)
Supplement: Supplementary Material [file imag_a_00102-supp.pdf]

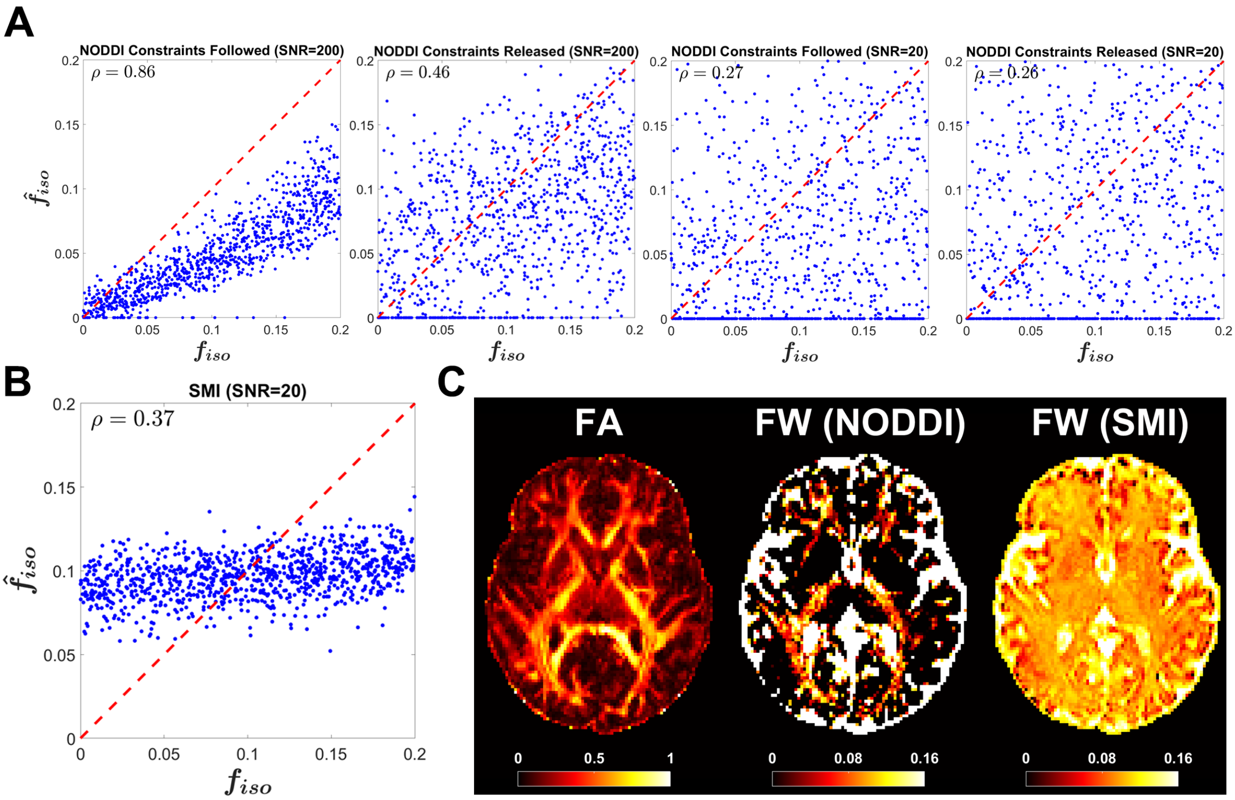

Figure S1: **Free water estimation with two-shell protocols using NODDI and SMI.** (**A**) NODDI estimate of free water volume fraction $\hat{f}_{iso}$ is plotted against the ground truth f_{iso} of synthetic data for SNR=20 and 200, and for NODDI diffusivity constraints followed and released. Correlation ρ between estimates and ground truth is indicated on each plot. (**B**) SMI estimate $\hat{f}_{iso}$ against the ground truth with SNR=20 and constraints released, same as the last plot in (**A**). The prior distribution of f_{iso} is uniform between 0 and 0.2. As a result, the SMI estimate $\hat{f}_{iso}$ is roughly near the prior mean 0.1 due to lack of sensitivity to the CSF compartment. (**C**) Exemplary parametric maps of a 43-year-old female control. Fractional anisotropy (FA) and free water volume fraction (FW) estimated by NODDI and SMI are presented. NODDI and SMI can distinguish fiber tracts and ventricles, but within a fiber tract, NODDI exhibits noisy estimates while SMI lacks contrast.

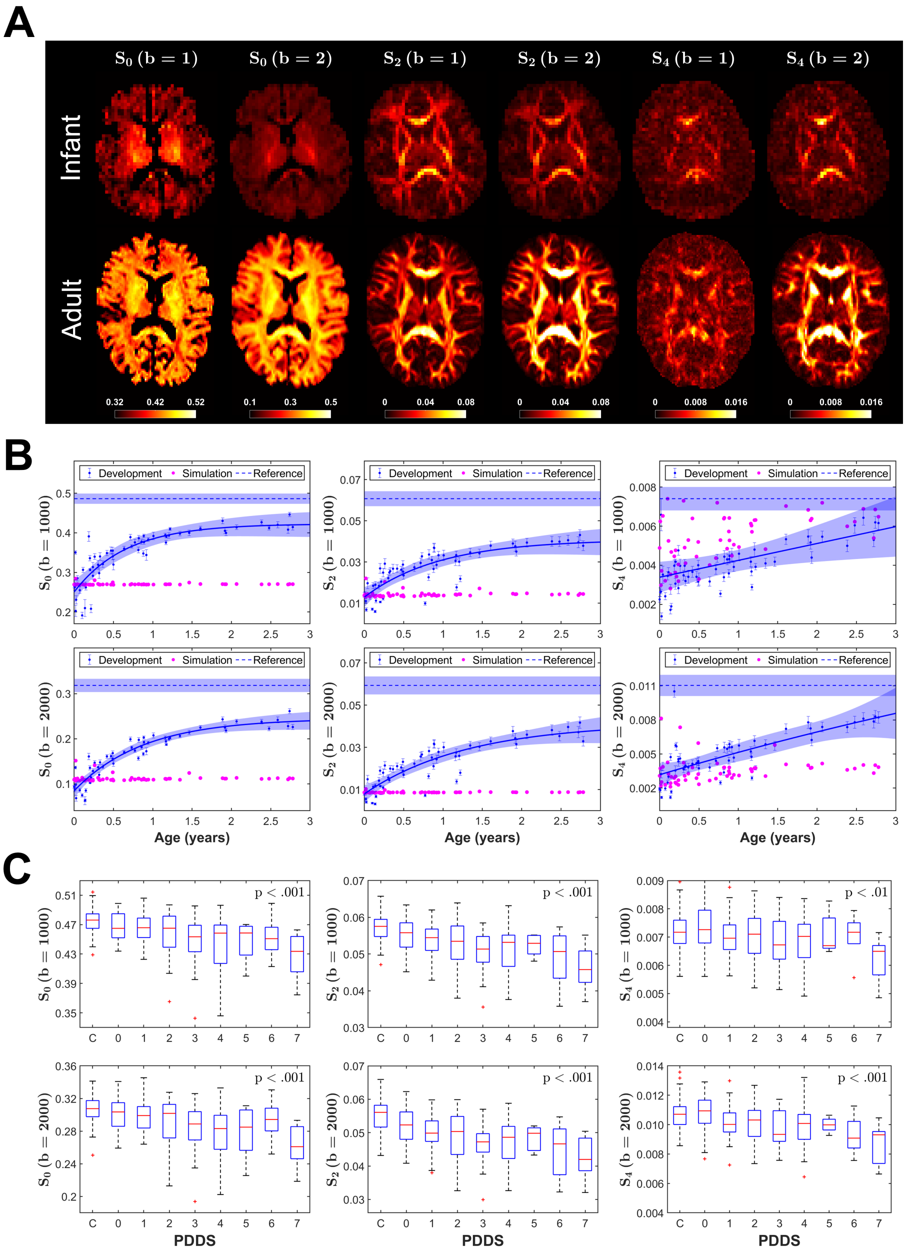

Figure S2: **Rotational invariants of the dMRI signals.** (**A**) Parametric maps of rotational invariants from a 3-month-old female infant and a 28-year-old female adult. b-value is in the unit of ms/μm^2. (**B**) To account for the SNR differences across subjects, the magenta dots represent the simulated rotational invariants of typical SM parameter combinations for newborn infants at the mean SNR level detected in each pediatric subject. In the trajectory of S_4 ($b = 1$) alone, which has a lower SNR than the rest of rotational invariants as shown in (**A**), the effect of varying SNR overtakes the change caused by development. (**C**) All rotational invariants of GCC mean excluding MS lesions (corrected for age) exhibit a significant decline (p-value indicated on the top right corner) as the severity of MS disability increases. On the leftmost position of the x-axis, 'C' stands for controls.

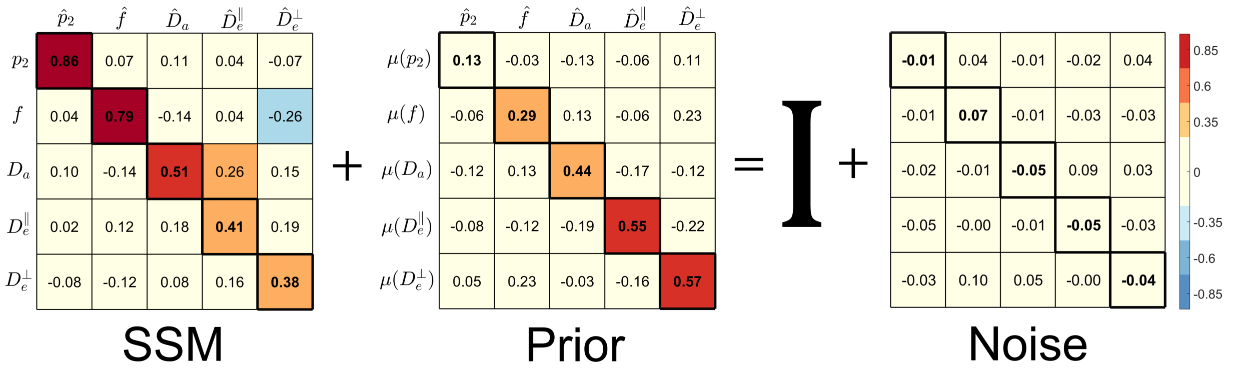

Figure S3: **Sensitivity-to-prior matrix.** Sensitivity-to-prior matrix was evaluated by applying linear regression to the prior mean of SMI to demonstrate the dependency of ML-based estimation on the prior. As is numerically validated in this figure, it is also possible to prove analytically that $S_{ij} + P_{ij} \approx I$ for a linear model estimated by a linear regressor, and this relationship becomes an approximation for a nonlinear model or regressor. This result suggests for ML-based estimators, deviations from the identity matrix in the SSM are related to the bias that is introduced to the estimator by the prior distribution (training set).

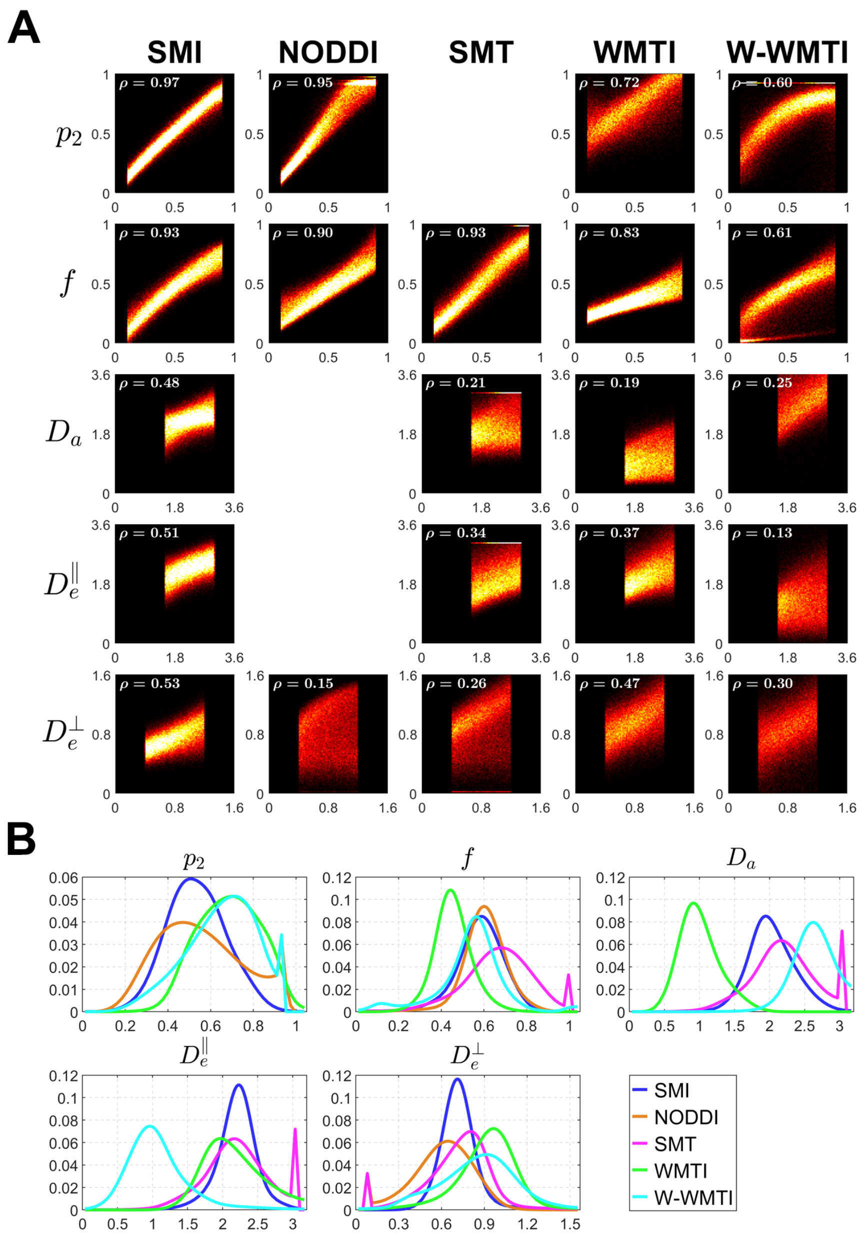

Figure S4: **Histograms of the SM parameter estimation.** (**A**) Simulations of realistic noise propagation for all estimators. Here, the estimates of each available SM parameter are plotted against the ground truth in scatter plots. Brighter color suggests higher data point density. Synthetic data are generated based on the SM for a two-shell protocol ($b = 1, 2$) and realistic signal-to-noise ratio SNR $= 25$ for $b = 0$. The Pearson correlation coefficient ρ between estimates and ground truth is indicated on each plot, where stronger correlations imply higher sensitivity. (**B**) Probability distributions of SM parameters from *in vivo* data. Distributions are made up of over 200,000 WM voxels from 177 young controls aged between 25 and 35 years old.

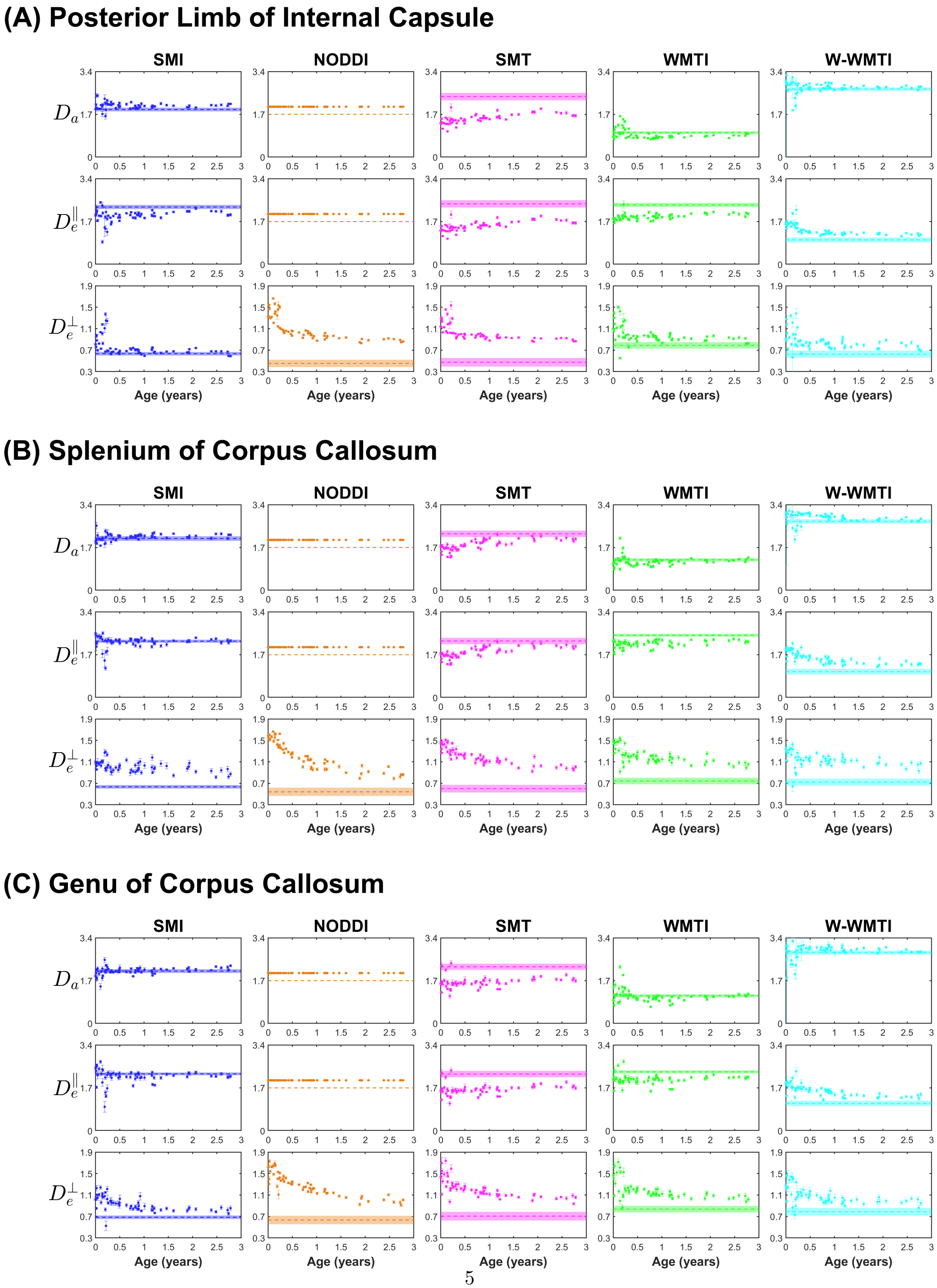

Figure S5: **Development trends of compartmental diffusivities in white matter ROIs.** Data points represent the ROI mean values for the pediatric subjects and the error bars indicate its 95% confidence interval. As a reference for the pediatric data, the dashed line and its neighboring shaded area represent the mean and standard deviation of the corresponding ROI mean for 177 controls aged between 25 and 35 years old.

Table S1: Conceptual comparison between WM estimators

Estimator Name	Compartments	Diffusivity Constraints	ODF	Independent parameters	Estimation Method
SMI	IAS + EAS	Unconstrained	Unconstrained	$p_2,\ f,\ D_a,\ D_e^{\|},\ D_e^{\perp}$	ML
NODDI	IAS + EAS + CSF	$D_a = D_e^{\|} = 1.7$, $D_e^{\perp} = D_e^{\|} \cdot (1-f)$	Watson distribution	$p_2,\ f,\ f_{iso}$	MLE
SMT	IAS + EAS	$D_a = D_e^{\|}$, $D_e^{\perp} = D_e^{\|} \cdot (1-f)$	Factored out by spherical mean	$f,\ D_a\ (D_e^{\|})$	MLE
WMTI	IAS + EAS	$D_a \leqslant D_e^{\|}$	Fibers more or less aligned	$p_2,\ f,\ D_a,\ D_e^{\|},\ D_e^{\perp}$	Analytically derived from DKI metrics
W-WMTI	IAS + EAS	$D_a \geqslant D_e^{\|}$	Watson distribution	$p_2,\ f,\ D_a,\ D_e^{\|},\ D_e^{\perp}$	Analytically derived from DKI metrics

Table S2: SMI time constants (in years) of development trajectories for WM regions

**WM region**	$\tau(f)$	$\tau(p_2)$
Cerebellar Peduncle	0.51 ± 0.38	0.10 ± 0.11
Genu Of Corpus Callosum	1.89 ± 1.12	0.18 ± 0.16
Body Of Corpus Callosum	1.20 ± 0.45	0.30 ± 0.22
Splenium Of Corpus Callosum	0.94 ± 0.27	0.34 ± 0.22
Anterior Limb Of Internal Capsule	0.72 ± 0.34	0.12 ± 0.12
Posterior Limb Of Internal Capsule	0.53 ± 0.16	0.18 ± 0.23
Anterior Corona Radiata	3.73 ± 5.70	0.14 ± 0.13
Superior Corona Radiata	1.06 ± 0.34	0.09 ± 0.14
Posterior Corona Radiata	0.92 ± 0.27	0.09 ± 0.08
Posterior Thalamic Radiation	1.26 ± 0.91	0.18 ± 0.16
Superior Longitudinal Fasciculus	1.33 ± 0.46	0.09 ± 0.12
